# β-Barrel Assembly Machinery (BAM) Complex as Novel Antibacterial Drug Target

**DOI:** 10.3390/molecules28093758

**Published:** 2023-04-27

**Authors:** Qian Xu, Min Guo, Feiyuan Yu

**Affiliations:** 1 Laboratory of Molecular Pathology, Department of Pathology, Provincial Key Laboratory of Infectious Diseases and Molecular Immunopathology, Shantou University Medical College, Shantou 515041, China; 2 Allergy Clinic, Zibo Central Hospital, Zibo 255000, China; 3 Department of Cell Biology and Genetics, Shantou University Medical College, Shantou 515041, China

**Keywords:** β-barrel assembly machinery, BAM complex, BamA, Gram-negative bacteria, drug resistance, drug target

## Abstract

The outer membrane of Gram-negative bacteria is closely related to the pathogenicity and drug resistance of bacteria. Outer membrane proteins (OMPs) are a class of proteins with important biological functions on the outer membrane. The β-barrel assembly machinery (BAM) complex plays a key role in OMP biogenesis, which ensures that the OMP is inserted into the outer membrane in a correct folding manner and performs nutrient uptake, antibiotic resistance, cell adhesion, cell signaling, and maintenance of membrane stability and other functions. The BAM complex is highly conserved among Gram-negative bacteria. The abnormality of the BAM complex will lead to the obstruction of OMP folding, affect the function of the outer membrane, and eventually lead to bacterial death. In view of the important role of the BAM complex in OMP biogenesis, the BAM complex has become an attractive target for the development of new antibacterial drugs against Gram-negative bacteria. Here, we summarize the structure and function of the BAM complex and review the latest research progress of antibacterial drugs targeting BAM in order to provide a new perspective for the development of antibiotics.

## 1. Introduction

The advent of antibiotics made great contributions to human life and health. However, the abuse of antibiotics has accelerated the evolution and reproduction of bacteria [1]. The emergence of methicillin-resistant *Staphylococcus aureus* (MRSA) has raised awareness of antibiotic resistance as a global crisis threatening human health [2]. The latest research shows that 4.95 million people died from antibiotic failure in 2019 alone [3]. In addition, the emergence of multidrug-resistant bacteria has exacerbated the severity of the problem. The World Health Organization predicts that in 2050, more than 10 million people will die from antibiotic resistance, and the death rate will even exceed that of cancer [4]. At the same time, the development of antibiotics has slowed down significantly. During the past decade, only a small number of novel antimicrobial drugs were approved by the FDA, and most were based on chemical structure modifications of existing drugs [5]. There are many reasons for this situation. On the one hand, pharmaceutical companies are reluctant to invest due to the high cost of antibiotic research and development but low returns; on the other hand, there is still a lack of effective antimicrobial drug targets. Therefore, finding the next generation of innovative antibacterial drug targets is the key to accelerating the development of novel antibiotics.

Bacteria can be divided into Gram-positive bacteria and Gram-negative bacteria according to Gram staining. Gram-positive bacteria have a thick and dense peptidoglycan layer in the cell wall [6], and Gram-negative bacteria have a thinner, lipid-rich peptidoglycan layer and an outer membrane layer outside the peptidoglycan layer. The aqueous space between the outer membrane and the cytoplasmic membrane (also known as the inner membrane) is called the periplasm [7,8]. The outer membrane is an asymmetric structure composed of the outer lipopolysaccharide, the inner phospholipid bilayer, and the outer membrane protein (OMP). It has the important functions of maintaining cell osmotic pressure, material transfer, and protecting the bacteria from external damage. It can also mediate Gram Negative bacteria produce intrinsic resistance [9,10].

Gram-negative bacterial OMPs have important biological functions due to their location. OMPs can act as channel proteins to transport nutrients, such as porin OmpF, OmpC, PhoE, etc. [11]. OMPs can also act as efflux pumps, which can efflux drugs and peptides and are closely related to bacterial multidrug resistance [12]. The function of OMPs also includes maintaining cell shape and outer membrane stability, which is related to the formation of peptidoglycan and pili assembly, such as the OmpA family [13,14,15]. In addition, some OMPs function as enzymes or act as adhesion factors in bacterial virulence, etc. [16]. The OMPs are almost all β-barrel proteins, which are barrel-shaped structures formed by an even number of antiparallel β-sheets through adjacent hydrogen bonds [17]. The β-sheet is connected by a long loop on the extracellular sides and a short loop on the periplasmic side [18]. The central space of the β-barrel OMPs structure is hydrophilic, and the outer surface is hydrophobic. The assembly of OMPs relies on β-barrel assembly machinery (BAM), which ensures that OMPs are inserted into the outer membrane in a correct folding manner and perform biological functions [19]. BAM is highly conserved in Gram-negative bacteria. Studies have shown that the BAM complex can improve the folding efficiency of OMPs, and the abnormality of BAM will lead to abnormal aggregation and precipitation of OMPs, eventually leading to bacterial death. This suggests that BAM can be used as a target for antibacterial drugs [8,20]. In this review, we first introduce the structure and function of the BAM complex and then review the latest research progress of antibacterial drugs targeting BAM.

## 2. The Structure of the BAM Complex

**Figure 1:**
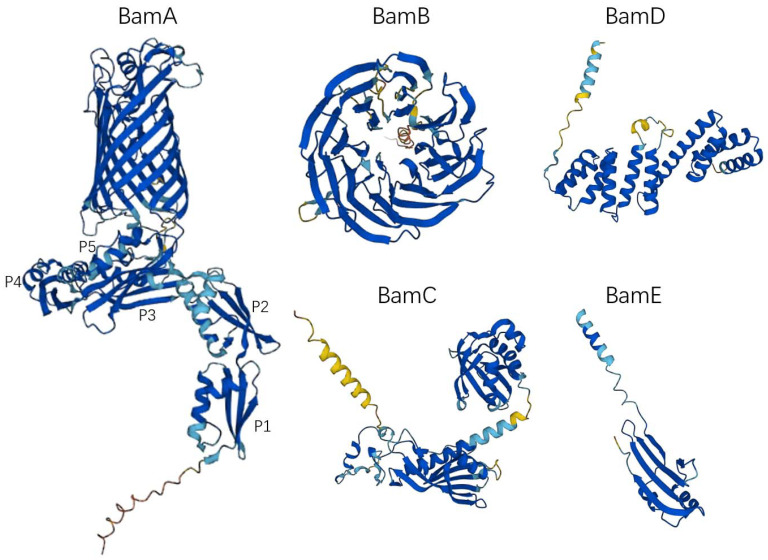
The monomer structure of the BAM complex. All structures were obtained from AlphaFold Protein Structure Database (https://alphafold.ebi.ac.uk, accessed on 11 December 2022). The UniProt IDs of the BAM complex molecules in Table 1 were used to access the 3D structure. P1, POTRA 1; P2, POTRA 2; P3, POTRA 3; P4, POTRA 4; P5, POTRA 5.

The BAM complex of most Gram-negative bacteria consists of the outer membrane protein BamA (Omp85/YaeT) and the lipoproteins BamB (YfgL), BamC (NlpB), BamD (YfiO), and BamE (SmpA) (Figure 1) [8]. BamA is the core component of the BAM complex, which is highly conserved in all Gram-negative bacterial species and plays an important role in the assembly of OMPs. Other lipoproteins such as BamB, BamC, BamD, and BamE directly or indirectly interact with BamA, anchored to the periplasmic face of the outer membrane [21]. For most Gram-negative bacteria, BamA and BamD are essential components of the BAM complex, and the deletion of BamB, BamC, and BamE affects the function of the BAM complex but does not cause bacterial death [22]. Table 1 shows the features of BAM complex molecules.

### 2.1. BamA

In 2003, people first discovered and confirmed the existence of BamA in *Neisseria meningitidis* and found that BamA plays an important role in the assembly of β-barrel OMP [23]. Subsequently, it was further clarified that BamA is essential in *Escherichia coli* (*E. coli)* [8]. The absence of BamA protein will lead to the misfolding of the β-barrel OMP, which cannot be inserted into the bacterial outer membrane correctly, resulting in damage to the outer membrane structure, and eventually leading to the death of the bacteria [24,25]. BamA is a member of the Omp85/TPS (outer-membrane protein of 85 kDa/two-partner secretion) superfamily and is also a class of OMPs. Its C-terminal contains a transmembrane β-barrel domain, and the N-terminal periplasmic domain contains a set of POTRA (polypeptide transport-associated, POTRA) domains related to polypeptide transport (Figure 1) [26]. The C-terminal β-barrel domain consists of 16 antiparallel β sheets; the first β sheet (β1) and the last β sheet (β16) form a barrel structure through hydrogen bonding [9,27]. The β-barrel can undergo lateral opening [28], which facilitates the folding and assembly of OMP. The POTRA domain is responsible for OMP substrate recognition, docking, and folding of OMPs [29]. BamA of most Gram-negative bacteria, including *E. coli*, has five POTRA domains, POTRA 1-5 from N-terminus to C-terminus [30]. The sequence similarity of each POTRA is low; however, the order of secondary-structure elements is usually β-α-α-β-β [31]. Among them, POTRA1-2 and POTRA3-5 are two rigid bodies, and the link between POTRA2 and POTRA3 is flexible. The POTRA domain can serve as a scaffold for BAM complex assembly, interacting with other lipoprotein components. Studies have shown that POTRA3 is connected to BamB; however, deleting either POTRA2-POTRA5 will result in the loss of BamB [32], whereas BamD is linked to BamA via POTRA5 [32]. In different bacterial species, the importance of each POTRA domain is different. In *N. meningitidis*, POTRA5 is an essential component of the OMP folding process, whereas mutations in the other four only affect the rate of folding [33]. In *E. coli*, mutant POTRA3-5 can affect the function of BamA and lead to bacterial death, while mutant POTRA1-2 will not cause bacterial death, but will affect the growth and reproduction rate of bacteria [32].

### 2.2. BamB

BamB consists of an eight-bladed β-propeller with four beta strands per blade (Figure 1), which is the largest lipoprotein component of the BAM complex [34]. BamB directly interacts with the PTOTRA3 domain of BamA [35,36], and it has also been found that PTOTRA2-4 interacts with BamB [37]. Depletion of BamB leads to increased misfolding of OMPs and reduces the number of proteins assembled into the outer membrane, but is not lethal [8]. However, the loss of BamB will change the permeability of the bacterial outer membrane [38], increase the sensitivity to antibiotics [39], and also lead to the weakening of the pathogenicity of some pathogens [39,40,41]. The specific role of BamB in OMP biogenesis is still unclear. SurA is a periplasmic chaperone that delivers outer membrane protein precursors to BamA. Previous studies have shown that the *E. coli* strains lacking SurA have the same phenotype as the strains lacking BamB, that is, the rate of OMP biogenesis is significantly decreased [42]. This suggests that BamB may promote the transfer of β-barrel OMP precursor to BamA. In addition, the protein structure of BamB contains WD40 repeat-like domains as a scaffolding protein to assist the PTOTRA domain to promote the folding of OMPs [37,43,44].

### 2.3. BamC

The BamC is a component of the β-barrel assembly machine in *E. coli*. BamC consists of two helix-grip domains connected by a flexible linker in the middle (Figure 1) [45]. The N-terminal unstructured region of BamC interacts with BamD and may be responsible for regulating the function of BamD [46]. Cells lacking BamC reduce folding and assembly rates of OMPs, but not lethally [47]. However, the deletion of BamC may affect outer membrane permeability and sensitize bacteria to the external environment [48]. It has been reported that the C-terminal domain of BamC is exposed on the surface of *E. coli*; however, whether BamC is transmembrane is still controversial [49,50].

### 2.4. BamD

BamD is an essential component of the BAM complex and is the most conserved lipoprotein in the BAM complex [22]. BamD is composed of 10 α-helices connected by loops to form five tetratricopeptide repeats (Figure 1) [51]. The C-terminal domain of BamD interacts with BamC and BamE to form BamCDE, which directly interacts with BamA [51]. The N-terminal domain of BamD can recognize and bind the C-terminal β-strand of the OMP, which is responsible for receiving unfolded OMPs and transferring them to the β-barrel domain of BamA for further folding and subsequent assembly into the outer membrane [51,52,53].

### 2.5. BamE

BamE is the lowest molecular weight lipoprotein found in the BAM complex and may exist as a dimer [54]. Similar to BamC, BamE is not an essential component of the BAM complex [47]. Studies have shown that loss-of-function mutations of BamE cause only minor OMP folding defects [47]. BamE contributes to the interaction between BamA and BamD, which is beneficial to maintain the stability of the BAM complex [47]. In addition, BamE may also be involved in cell wall maintenance [54].

## 3. Folding and Insertion of OMPs by the BAM Complex

**Figure 2:**
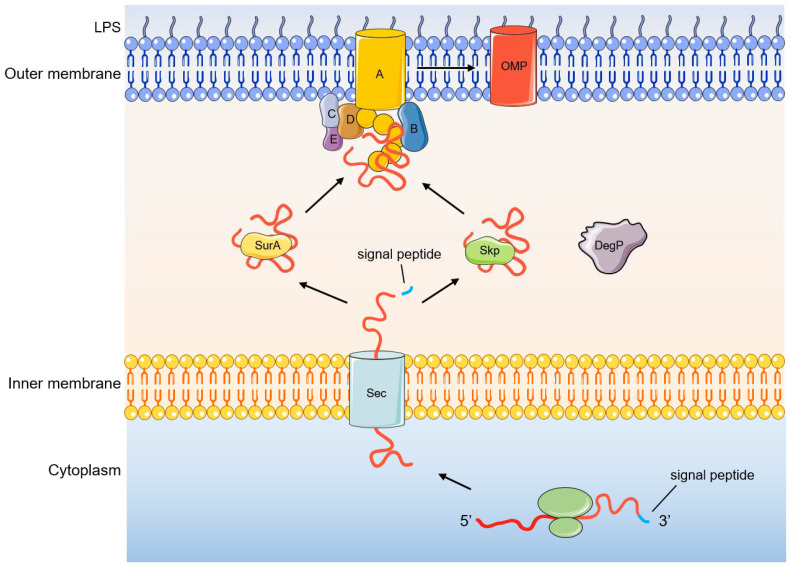
Process of OMP biogenesis.

The OMP is first translated on the ribosome into a nascent polypeptide that does not have biological functions, that is, OMP precursors, and its N-terminus has signal peptides. The signal peptide can be recognized by the Sec translocation system, and pass through the inner membrane to the periplasmic space (Figure 2) [55,56,57,58,59]. Next, signal peptidase recognizes and excises the signal peptide at the N-terminus of the OMP precursor. The OMP precursor is then transported to the BAM complex by the molecular chaperone SurA or DegP/Skp in the periplasm to complete the final folding and membrane insertion [18]. SurA belongs to the peptidylprolyl isomerase family and plays a role as a molecular chaperone in the biogenesis of OMPs [60,61]. SurA can directly interact with BamA and the depletion of SurA will lead to a decrease in the density of the outer membrane and a decrease in the correct folding and assembly of OMPs [62]. Skp acts as a chaperone that binds unfolded OMPs in a trimer form [63]. DegP functions as a molecular chaperone at low temperatures and exhibits proteolytic activity at elevated temperatures [64,65]. However, recent studies have also shown that DegP only acts as a protease to degrade misfolded OMPs and has almost no role as a molecular chaperone [66]. Deletion of Skp/DegP does not alter outer membrane density but leads to the accumulation of toxic misfolded proteins and activates the σ^E^ stress response [62]. In general, OMPs are mostly transported through SurA, which is the main transport pathway, and when SurA is lacking, they can also be transported through the DegP /Skp pathway [67,68,69]. These two transport pathways are parallel pathways, and when one of the transport pathways loses function, it does not lead to synthetic lethal [62,70].

**Figure 3:**
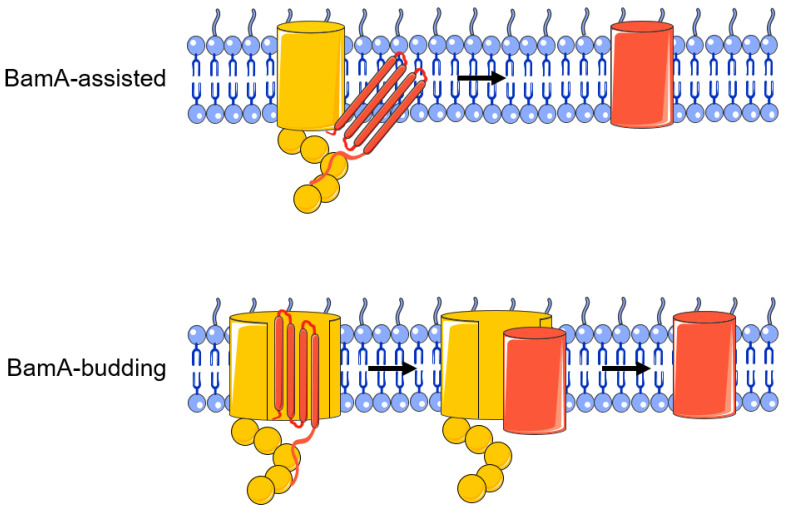
BamA-assisted model and BamA-budding model.

The mechanism of BamA-mediated assembly of OMP folds is unclear, and there are two main theories (Figure 3) [71]. The first is the “BamA-assisted” model. β-barrel proteins have folding-free energies [72,73]. Molecular dynamics simulations show that some OMPs can spontaneously fold or partially fold on the periplasmic side [74,75]. BamA mainly plays a supporting and auxiliary role; it may cause a membrane defect and disrupt the homeostasis of the outer membrane and promote the completion of the membrane insertion process of OMPs [9]. In this process, whether the conformation of BamA changes or not is still controversial. The second is the “BamA-budding” model. The hydrogen bond connection between the N-terminal 1st β-strand and the C-terminal 16th β-strand of the BamA barrel structure is weak, resulting in the breakage of the hydrogen bond and the formation of a “lateral gate” [28]. After the OMP substrate enters the BamA barrel structure, the “lateral gate” of the β-fold barrel opens to expose unpaired β-strands, which can serve as folding templates for the OMP. Therefore, the combination of the above two proteins will form a BamA-OMP intermediate [28,76]. When OMP completes folding, its β-barrel domain closes, and OMP buds off the BamA and inserts into the outer membrane [9,69]. At present, more research results support the BamA-assisted model rather than the budding model [9].

## 4. Research Progress of Drugs Targeting the BAM Complex

The outer membrane is an important barrier for Gram-negative bacteria to resist the external environment. The functions of OMPs include nutrient uptake, cell adhesion, antibiotic resistance, and maintenance of membrane selective permeability, etc. [69]. The BAM complex is responsible for the organization of OMP folding and is highly conserved among Gram-negative bacteria. With in-depth understanding of the structure and function of BAM, BAM has shown considerable potential as an antimicrobial drug target for Gram-negative bacteria. Among them, BamA and BamD, as important components of BAM, are conserved in most Gram-negative bacteria and are currently popular targets in the development of anti-Gram-negative bacteria drugs.

### 4.1. Darobactin

**Figure 4:**
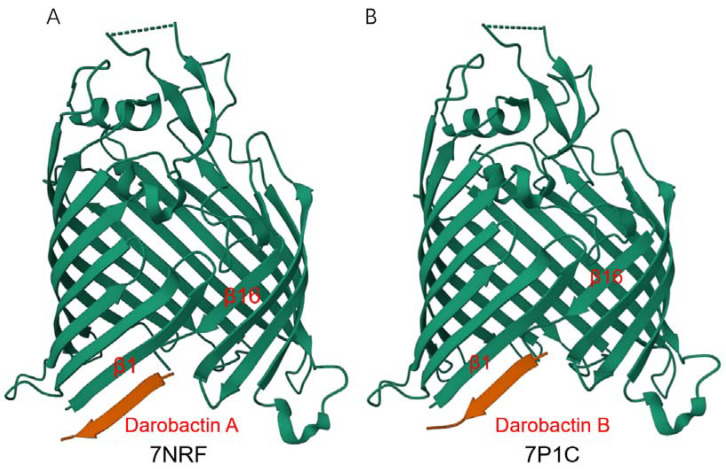
Crystal structure of *E.coli* BamAβ-barrel in complex with darobactin. Structures of darobactin A (**A**) and darobactin B (**B**) were obtained from RCSB PDB (https://www.rcsb.org/, accessed on 23 February 2023).

Darobactin A is a heptapeptide (Trp1-Asn2-Trp3-Ser4-Lys5-Ser6-Phe7) antibiotic produced by *Photorhabdus* [77], which exhibits good antibacterial activity against Gram-negative bacteria but has no effect on Gram-positive bacteria. Imai et al. obtained mutant strains highly resistant to darobactin A by repeatedly culturing with progressively increasing darobactin A. Sequencing of the mutant strains revealed a mutation in the gene encoding BamA and finally confirmed that BamA is the target of darobactin A. Darobactin A can specifically bind to BamA, keep the BamA lateral gate closed, and prevent substrates from entering the BAM complex for folding and membrane insertion. Within 24 h after mice were infected with pathogens (*E. coli*, *K. pneumoniae*, and polymyxin-resistant *P. aeruginosa*), all mice given a single dose of darobactin A survived, while all mice in the control group died [78]. Further research found that darobactin A binds to the lateral gate of BamA in an antiparallel β sheet conformation: Trp1 of darobactin A pairs Ile430 of BamA to form backbone hydrogen bonds, continuing all the way to Phe7, which pairs with Gly424 (Figure 4A), and is a competitive inhibitor of the combination of OMP β-signal (a conserved sequence motif found at the C-terminus of OMP) and BamA β1 strand [79]. Darobactin A only plays a role in the initial stage of OMP folding, and when OMP β signaling is stably combined with BamA β1 strand, darobactin A loses its inhibitory effect [80]. Darobactin A is a particularly promising candidate for Gram-negative bacteria inhibition, and the synthesis and structure optimization of its derivatives has become a hotspot in the field of antibacterial drug research [81,82,83,84,85]. Darobactin B (Figure 4B) displayed comparable or even better activities against clinically relevant Gram-negative pathogens [81]. Dynobactin is a novel peptide antibiotic obtained by searching for genes distantly related to the darobactin operon. Although it is different from darobactin in structure, it also binds to BamA lateral gate and exhibits a good antibacterial effect in vivo [77].

### 4.2. JB-95

Murepavadin is a peptidomimetic antibiotic targeting *Pseudomonas*. The antibiotic can bind to β-barrel protein LptD, interfere with the assembly of LPS, and affect outer-membrane biogenesis [86]. Murepavadin has shown strong antibacterial effects in in vivo and in vitro experiments and clinical trials [86,87,88,89]. In 2015, the team that developed Murepavadin reported a conformationally constrained hairpin peptidomimetic (called JB-95), which exhibited strong antibacterial activity against Gram-positive and Gram-negative bacteria, especially *E. coli*. JB-95 does not affect the expression of BamA, but interacts with BamA, disrupts the outer membrane structure, and leads to the death of bacteria [90]. In 2019, the same team screened murepavadin-related cyclic peptides for Gram-negative ESKAPE pathogens and obtained some peptides that can resist colistin-resistant strains. These peptides were chimerized with peptide macrocycle of polymyxin B (PMB) and colistin to obtain new compounds. In the antibacterial activity test of drug-resistant bacteria, these compounds performed well and showed favorable safety and pharmacokinetic properties. By high-resolution nuclear magnetic resonance (NMR) spectroscopy, it was found that these compounds combined with extracellular loops (L4, L6, and L7) of BamA to stabilize BamA in the closed state. However, whether these compounds lead to cell permeabilization and death by inhibiting the foldase activity of the BAM complex, or enhance the antibacterial effect of polymyxin macrocycle by binding to BamA, remains to be further studied [91].

### 4.3. LlpAs

**Figure 5:**
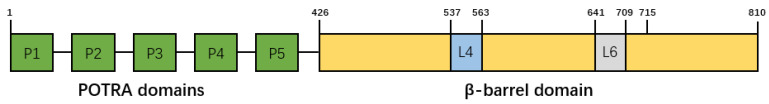
Schematic structure of *E. coli* BamA. The N-terminal of BamA contains five POTRA domains: P1, P2, P3, P4, and P5 (green); and the C-terminal of BamA is a β-barrel domain (yellow). LlpAs and MAB1 target extracellular loop 6 (grey) and loop 4 (blue), respectively.

Bacteria secrete bacteriocins, which are used to kill competing microorganisms. Pseudomonads secrete multiple bacteriocins for better colonization in diverse environments. Among them, Lectin-like bacteriocins, also called LlpAs, contain a tandem of B-lectin domains. The carboxy-terminal domain binds to the D-rhamnose residues of LPS, and the amino-terminal domain acts on BamA through the extracellular loop 6 (L6, Figure 5), interfering with the assembly of OMPs [92]. In order to avoid self-inhibition, the LlpAs secreted by Pseudomonads do not target their own L6 because the L6 of BamA expressed by Pseudomonads is different from the target [92]. However, how LlpA binds to BamA through L6 at the molecular level is still unclear. LlpB is a subclass of Pseudomonas lectin-like proteins excavated through genome mining, which also exhibits bactericidal activity. Whether LlpB functions through BamA remains to be investigated [93].

### 4.4. MRL-494

Hart et al. discovered MRL-494, an accidentally synthesized compound while screening for compounds not affected by the outer membrane barrier and efflux pumps [94]. Cellular thermal shift assay (CETSA) showed that MRL-494 targets BamA, but not other members of BAM [94]. MRL-494 cannot penetrate the outer membrane of bacteria and acts only on the cell surface [94]. Although its specific site of action is not yet clear, the characteristic of MRL-494 acting on the cell surface prevents it from being affected by intrinsic drug resistance and efflux pumps. For Gram-positive bacteria lacking an outer membrane, MRL-494 can exert an antibacterial effect by destroying the cytoplasmic membrane [94]. However, some recent studies have found that MRL-494 can disrupt the outer membrane of bacteria, and, combined with rifampicin, can play a synergistic activity against a variety of Gram-negative bacteria [80,95].

### 4.5. MAB1

MAB1 is a BamA-targeted monoclonal antibody that specifically binds to BamA in *E. coli*, but not other Gram-negative bacteria. It antagonizes the folding activity of OMP by directly binding to BamA extracellular loop 4 (L4, Figure 5), which is a surface-exposed BamA epitope. Alterations in the structure and outer membrane fluidity of LPS can affect the antibacterial effect of MAB1 [96,97].

### 4.6. IMB-H4

Li et al. confirmed the interaction between BamA and BamD by Yeast Two-Hybrid Assay, and the compound IMB-H4 was screened by a yeast two-hybrid screening system [98]. IMB-H4 can bind to BamA, interfere with the interaction between BamA and BamD, and destroy the integrity of the outer membrane. IMB-H4 exhibits potent antibacterial activity and enhances the activity of other antibiotics by disrupting outer membrane permeability [98].

### 4.7. FIRL

FIRL (Phe-Ile-Arg-Leu-CONH(2)) is a peptide synthesized based on homologous sequences of BamD, which can competitively bind to BamA of *P. aeruginosa*. FIRL can affect the outer membrane permeability of *P. aeruginosa* but has no bactericidal effect. The combination of FIRL with other antibiotics can enhance antimicrobial activity [99].

### 4.8. BamD Inhibitory Peptide

BamA and BamD are indispensable components in the BAM complex. Studies have found that BamD can interact with unfolded BamA and promote the assembly of BamA [52]. BamA is a protein of 810 amino acids (Figure 5). Hagan et al. divided unfolded BamA into four segments and found that BamD specifically bound the C-terminal of BamA (715–810 fragment) and can inhibit the folding of the BAM complex [53]. Then, they found that the inhibitory effect of the above polypeptide was attributed to residues at positions 769–776, and the expression of fragments containing residues 715–810 of BamA would lead to growth defects and sensitizes *E. coli* to vancomycin and rifampicin [53].

### 4.9. NTZ

Nitazolamide (NTZ), a small molecule, is a clinically used antiparasitic drug. Studies have found that NTZ can interfere with the folding of the usher protein in the outer membrane and thus inhibit the biological occurrence of bacterial pili [100,101]. NTZ targets BAM and specifically interferes with the folding of usher proteins without affecting other OMPs. This process requires the participation of lipoproteins BamB, BamD, and BamE [102].

### 4.10. VUF15259

Anti-virulence drugs targeting virulence factors are emerging strategies for antibiotic development [103]. The autotransporter (AT) pathway is the main pathway for the secretion of virulence factors in a variety of Gram-negative bacteria. Inhibition of this pathway can achieve a bacteriostatic effect. The compound VUF15259, obtained through transcriptomic analysis and screening, is the first reported inhibitor of the AT secretion pathway. VUF15259 interferes with the insertion of β-barrel OMP into the outer membrane; however, whether it affects the function of the BAM complex is unknown [104].

As shown in Table 2, the inhibitors acting on the BAM complex were all discovered in the past decade, and most of the BAM complex inhibitors target BamA. Among these inhibitors, five are peptides (darobactin/dynobactin, JB-95, LlpAs, FIRL, and BamD Inhibitory Peptide), four are small molecules (MRL-494, IMB-H4, NTZ, and VUF15259), and one is a monoclonal antibody (MAB1). Among the BAM inhibitors currently studied, the interaction site between darobactin and BamA is clear with resolved crystal structure (Figure 4), and the structure of the interaction between other inhibitors and BamA remains to be studied. Furthermore, studies have found that nanobodies stabilized BamA to form two stable conformations (with open or closed BamA lateral gate), which might be valuable for the understanding of the BamA-assisted outer membrane protein insertion mechanism and for developing improved antibiotics.

## 5. Conclusions and Prospects

Antibiotic resistance poses a serious challenge to the treatment of antibacterial infections. One of the solutions is to develop novel antibiotics by finding effective drug targets. The outer membrane is a unique structure of Gram-negative bacteria, which can protect the bacteria from the external environment. Since the discovery of BamA in *Neisseria meningitidis* in 2003, studies have shown that the BAM complex is a key molecule in OMP biogenesis of Gram-negative bacteria and is expected to be a potential candidate for a drug target. The BAM complex consists of the OMP BamA and the lipoprotein BamB-E. Among them, BamA and BamD are essential components and are highly conserved in Gram-negative bacteria. Therefore, most of the known BAM inhibitors target BamA or BamD.

Although BamA or BamD inhibitors have not been used clinically, they have shown good antibacterial potential. However, the role of each component of the BAM complex has not been fully elucidated, and how the BAM complex promotes the folding and insertion of OMPs is still unclear. In recent years, with the application of X-ray crystallography and cryo-electron microscopy, the structure and function of the BAM complex have become increasingly clear. Therefore, in the past five years, there have been gradual reports of inhibitors targeting the BAM complex. However, due to the complexity of the structure and function of the BAM complex, the development of antibacterial drugs targeting BAM is still limited. With the in-depth research on the structure and function of the BAM complex, and the vigorous development of technologies such as characterization analysis, gene sequencing, and artificial intelligence, the potential of the BAM complex as a drug target will be further explored to promote the development of novel antibiotics.

## Figures and Tables

**Table 1:** Features of BAM complex molecules (*Escherichia coli*, strain K12).

Name	Also Known As	Length (Amino Acids)	Mass (Da)	Identifier (Uniprot)
BamA	ecfK; ECK0176; yaeT; yzzN; yzzY	810	90,553	P0A940
BamB	ECK2508; yfgL	392	41,887	P77774
BamC	dapX; ECK2473; nlpB	344	36,842	P0A903
BamD	ecfD; ECK2593; yfiO	245	27,829	P0AC02
BamE	b2617, ECK2613, smpA, smqA	113	12,302	P0A937

**Table 2:** BAM inhibitors and their characteristics.

Inhibitors	Published Year	Molecular Target	Type	Reference
Darobactin A	2019	BamA (lateral gate)	peptide	[78]
Darobactin B	2021	BamA (lateral gate)	peptide	[81]
Darobactin 9	2021	BamA (lateral gate)	peptide	[82]
Darobactin 22	2022	BamA (lateral gate)	peptide	[84]
Dynobactin A	2022	BamA (lateral gate)	peptide	[77]
JB-95	2015	BamA, LptD	peptide	[90]
		BamA(extracellular loops 4, 6 and 7 of BamA)		
LlpAs	2018	BamA (extracellular loop 6)	peptide	[105]
MRL-494	2019	BamA	small molecule	[94]
MAB1	2018	BamA (extracellular loop 4)	monoclonal antibody	[96]
IMB-H4	2020	BamA	small molecule	[98]
FIRL	2012	BamA	peptide	[99]
BamD Inhibitory Peptide	2015	BamD	peptide	[53]
NTZ	2019	BAM complex	small molecule	[102]
VUF15259	2019	BAM complex	small molecule	[104]

## Data Availability

Not applicable.
